# Identification of a major locus interacting with *MC1R* and modifying black coat color in an F_2_ Nellore-Angus population

**DOI:** 10.1186/1297-9686-46-4

**Published:** 2014-01-24

**Authors:** Lauren L Hulsman Hanna, James O Sanders, David G Riley, Colette A Abbey, Clare A Gill

**Affiliations:** 1 Department of Animal Science, Texas A& M University, College Station, TX 77843, USA; 2 Current Address: Department of Animal Science, North Dakota State University, Fargo, ND 58108, USA

## Abstract

**Background:**

In cattle, base color is assumed to depend on the enzymatic activity specified by the *MC1R* locus, i.e. the extension locus, with alleles coding for black (*E*^*D*^), red (*e*), and wild-type (*E*^*+*^). In most mammals, these alleles are presumed to follow the dominance model of *E*^*D*^ > *E*^*+*^ > *e*, although exceptions are found. In *Bos indicus* x *Bos taurus* F_2_ cattle, some *E*^*D*^*E*^*+*^ heterozygotes are discordant with the dominance series for *MC1R* and display various degrees of red pigmentation on an otherwise predicted black background. The objective of this study was to identify loci that modify black coat color in these individuals.

**Results:**

Reddening was classified with a subjective scoring system. Interval analyses identified chromosome-wide suggestive (*P* < 0.05) and significant (*P* < 0.01) QTL on bovine chromosomes (BTA) 4 and 5, although these were not confirmed using single-marker association or Bayesian methods. Evidence of a major locus (*F* = 114.61) that affects reddening was detected between 60 and 73 Mb on BTA 6 (Btau4.0 build), and at 72 Mb by single-marker association and Bayesian methods. The posterior mean of the genetic variance for this region accounted for 43.75% of the genetic variation in reddening. This region coincided with a cluster of *tyrosine kinase receptor* genes (*PDGFRA*, *KIT* and *KDR*). Fitting SNP haplotypes for a 1 Mb interval that contained all three genes and centered on *KIT* accounted for the majority of the variation attributed to this major locus, which suggests that one of these genes or associated regulatory elements, is responsible for the majority of variation in degree of reddening.

**Conclusions:**

Recombinants in a 5 Mb region surrounding the cluster of *tyrosine kinase receptor* genes implicated *PDGFRA* as the strongest positional candidate gene. A higher density marker panel and functional analyses will be required to validate the role of *PDGFRA* or other regulatory variants and their interaction with MC1R for the modification of black coat color in *Bos indicus* influenced cattle.

## Background

Coat color phenotypes are used in breed recognition for cattle and other livestock species. Marketing strategies, such as the Certified Angus Beef program in the United States of America that only accepts cattle with at least 51% black coat color (and which exhibit Angus influence), have opened venues for price discrimination due to such qualifications. Availability of either premiums or discounts due to coat color phenotypes can have a direct impact on producers’ breeding strategies and an understanding of the genes and mechanisms involved in cattle pigmentation is desirable.

The melanocortin-1 receptor (alpha melanocyte stimulating hormone receptor, MC1R) is responsible for the primary switch of pheomelanin (red to yellow pigment) to eumelanin (black to brown pigment) in the melanogenesis pathway. In cattle, differences in base color are attributed to mutations in the *MC1R* gene, historically termed the extension locus, with alleles coding for black (*E*^*D*^), red (*e*), and wild-type (*E*^*+*^). These alleles were presumed to follow a dominance model, in which *E*^*D*^ > *E*^*+*^ > *e*. The wild-type allele (*E*^*+*^) produces a functional receptor that responds to both the α-melanocyte-stimulating hormone (α-MSH) ligand and its antagonist, agouti-signaling protein (ASP). The *E*^*D*^ allele, caused by a leucine to proline point mutation, creates a constitutively active receptor that results in eumelanin production most likely because of α-MSH ligand binding mimicry [1]. The recessive allele (*e*) leads to the production of only pheomelanin because of a frameshift mutation that creates a prematurely terminated, non-functional receptor [2,3].

Various degrees of red pigmentation were observed in ½ *Bos indicus* (Nellore) ½ *Bos taurus* (Angus) cattle with the *E*^*D*^*E*^*+*^ genotype at *MC1R*, which were expected to be black. This observation plus anecdotal evidence from other cattle crosses and mouse studies [4] suggest that other loci can modify the expected black coat color. The objective of this study was to identify loci that modify black coat color in F_2_ Nellore-Angus cattle heterozygous (*E*^*D*^*E*^*+*^) for *MC1R.* We hypothesized that degree of reddening on a black background was a quantitative trait controlled by multiple genes of small effect.

## Methods

Cattle used in this study were a subset of an experimental population, known as the Texas A&M McGregor Genomics Cycle 1 Population, located in central Texas (latitude: 31.3865, longitude: -97.4105) that consisted of 14 full-sibling F_2_ families produced by multiple ovulation and embryo transfer (ET) and founded by Nellore grandsires and Angus granddams. Blood samples were collected on all live-born animals in the population, and DNA was extracted from white blood cell pellets by proteinase K digestion. Parentage was assigned using a panel of 12 to 14 microsatellite markers. All procedures that involve animals were approved by the Texas A&M Institutional Care and Use Committee; AUP 2002–116, 2005–147 and 2008–234.

Calves were photographed at birth, steers were photographed in the feeding pens and females were photographed shortly after each calving. Because there was a wide spectrum of base colors observed in the population, all calves were genotyped for *MC1R* (RefSeq NM_174108.2) using the E3 and E4 primers designed by Klungland et al. [2], which amplifies a 739 base pair fragment of *MC1R* encompassing the SNP c.296C > T (*E*^*+*^ to *E*^*D*^ allele) and the deletion c.311delG (*e* allele).

**Figure 1:**
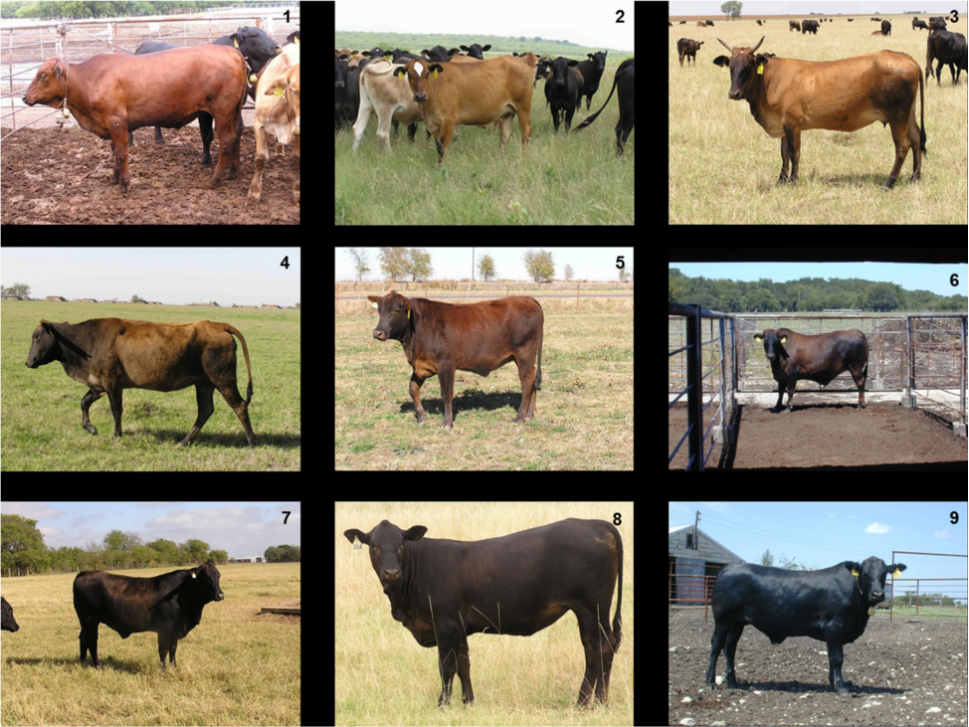
**Degree of black scoring system.** Examples of *E*^*D*^*E*^*+*^ F_2_ Nellore-Angus cattle that scored a 1 through 9, respectively, starting at the top left and proceeding left to right down the rows.

Comparison of resultant genotypes with photographs revealed that *E*^*D*^*E*^*D*^ homozygotes were black and *E*^*+*^*E*^*+*^ homozygotes ranged in color from yellow to black as expected. However, *E*^*D*^*E*^*+*^ heterozygotes often exhibited various degrees of reddening and were not black as predicted by the dominance series *E*^*D*^ > *E*^*+*^ > *e*. Degree of black was classified from 1 (lightly black; mostly red) to 9 (solid black) from photographs by three evaluators and a consensus score was determined (Figure 1). Photographs taken either in the feedlot or pasture at weaning age or older were available for 213 of the 238 *E*^*D*^*E*^*+*^ ET F_2_ cattle (four bulls, 115 steers, and 94 females).

Cattle influenced by *Bos indicus* breeds and *Bos indicus* often darken during the fall and winter although this has not been documented experimentally. Some photographs were taken in the spring/summer whereas others were taken in the fall/winter, which may introduce bias because of lighting and seasonal effects. Because of this, dates of images, which were available for all photographs of the project, and season of photographs, were constructed as a two-level fixed effect to reduce bias. Photographs were classified as summer if taken from May to October and winter if taken from November to April due to the seasonal changes in Texas. To avoid further bias, each photograph was scored separately and independently. No comparisons were made between photos.

To verify that a mutation in *MC1R* of *Bos indicus* origin did not contribute to the reddening phenotype, the MC1-RF2 and MC1-RR1 primers from Graphodatskaya et al. [5] were used to characterize the entire coding sequence of *MC1R* in a subset of the ET F_2_ and paternal half-siblings to the F_2_ animals (n = 31) produced by natural service that varied in their degree of black and their *MC1R* genotype. Straightbred *Bos indicus* and *Bos taurus* cattle and F_1_*Bos indicus* × *Bos taurus* cattle (n = 27) were also sequenced to further characterize the SNPs identified.

General linear model procedures of SAS® (SAS® Institute Inc., Cary, NC) were then used to evaluate fixed effects of sire, family-nested within sire, sex, year born or birth year-season combinations (four birth years, fall and spring seasons), and season photographed. The four bulls present in the dataset were excluded from subsequent analyses since they all ranged from 7 to 9 for degree of black and because color descriptions of bulls have shown that they can be darker than steers [6], which may create bias. Furthermore, one family consisted of only one individual. This individual was highly reddened, which could cause statistical bias when including family-nested within sire effects, and was therefore excluded from further analyses, leaving a total of 208 *E*^*D*^*E*^*+*^ ET F_2_ cattle for this study.

Neither birth year-season nor year of birth, when evaluated independently of each other, contributed to variation in degree of black (*P* > 0.25), and were excluded from the final model. No interactions of sex, sire, and family-nested within sire were detected. Residuals from the final model, which included sire, family-nested within sire, sex and season photographed were used for interval and association analyses and full models were used to run a Bayesian association analysis. Genotypes for these analyses were produced using the BovSNP50v1 assay (Illumina Inc., San Diego, CA) and SNP coordinates from build Btau4.0 of the bovine genome sequence. Data were filtered to remove genotypes for SNPs with a minor allele frequency less than 0.05, SNPs with completion rates below 90%, and SNPs that deviated from Hardy-Weinberg equilibrium (*P* < 0.0001). Initially, a set of 7112 markers informative in every family was used for interval analyses by applying the full sibling additive and dominance models of GRID*QTL* software [7], with 1 cM steps assuming 1 cM = 1 Mb, chromosome-wise significance thresholds established by 5000 permutations of the data, and the 95% confidence interval was established by 5000 bootstraps with resampling. Single-marker association analyses were performed using PLINK [8] for 39 480 SNPs with Bonferroni correction for multiple tests.

Both interval and single-marker association analyses are known to not estimate the size of QTL effects precisely [9,10], therefore Bayesian association analyses, which fit all available markers simultaneously, were also implemented using GenSel [11] to verify findings and provide more precise estimates of QTL effects. Based on preliminary analyses, the proportion of markers that did not contribute to variation in degree of black (designated as π) was known to be large, and therefore only a small subset of the markers (1 – π) was needed for inclusion in the model for Bayesian analysis. The exact proportion of those markers, however, was unknown. The π parameter was estimated by first calculating the genomic heritability estimate ($h_{g}^{2}$, a ratio of the posterior means of the genetic and phenotypic variances) when π = 0 using BayesC [12,13] procedures (i.e., all markers share a constant genetic variance and are included in the model) and then increasing π in intervals while keeping other parameters constant until a slight drop (0.01 to 0.02) in genomic heritability was observed, which determined π^. The architecture of this trait also indicated that a single variance component for all markers was inappropriate, so BayesB [14] procedures (i.e., all markers have individual variances) were applied with 51 000 iterations (1000 burn-in) using π^. Estimates of marker variance were grouped into megabase (Mb) regions using the “windowBV” option and association was determined using the posterior probability of association (PPA_*w*_) for each window [15,16]. Windows that accounted for greater than 0% of the genetic variance were ranked and those windows in the top 25^th^ percentile (i.e., PPA_*w*_ > 0.75) were said to be associated.

Coordinates of QTL were used to identify candidate genes concordant with the QTL region based on the Btau4.0 sequence assembly and genome maps at the National Center for Biotechnology Information (NCBI; http://www.ncbi.nlm.nih.gov). Genes within 5 Mb surrounding the QTL position were investigated based on available information in the NCBI database and reported results of other experiments. Functional homologs in mouse were also investigated.

Two additional sets of degree of black score residuals were generated. Haplotypes that spanned 1 Mb and were centered on a positional candidate gene relative to identified QTL were recovered. SNPs located within this 1 Mb region and that passed all previously described quality editing were phased with fastPHASE software [17]. Resolved haplotypes were tracked through the three-generation pedigree. First, breed-of-origin of each haplotype was assigned (Nellore versus Angus) and ordered based on parent of origin (sire then dam) resulting in four levels (Angus (AA), Angus-Nellore (AN), Nellore-Angus (NA), and Nellore (NN)). Second, unique haplotypes were numbered and paired in order of parent of origin to form phased genotypes of each animal. In each case, these scores were included as a fixed effect in the previously described models to produce a second and third set of residuals for interval analysis. For Bayesian analysis, breed-of-origin genotypes were included as a fixed effect in the previously described models to use for association analysis.

## Results

Two coding SNPs of *Bos indicus* origin were identified at c.583C > T (ss784304484) and c.663 T > C (ss784304485) of *MC1R*. These were the only additional coding variants that we identified in this intron-less gene and they were in complete phase with the *Bos indicus E*^*+*^ allele (c.296C > T). Detection of this single haplotype (TTC) in *Bos indicus* straightbred and crossbred cattle (n = 58) suggested that nucleotide variability in *MC1R* derived from *Bos indicus* did not contribute to variation in degree of black in *E*^*D*^*E*^*+*^ heterozygotes. Furthermore, we observed long-range haplotypes (due to linkage) that extended for more than 1 Mb either side of *MC1R*. All *E*^*D*^*E*^*+*^ heterozygotes were imputed from the haplotypes of their parents to be heterozygous for the CCT/TTC *MC1R* haplotypes. Within families, heterozygotes that shared the same pair of long-range haplotypes varied widely in their degree of black coat color; therefore, we think it is unlikely that a regulatory variant associated with MC1R could explain the observed reddening effect.

**Figure 2:**
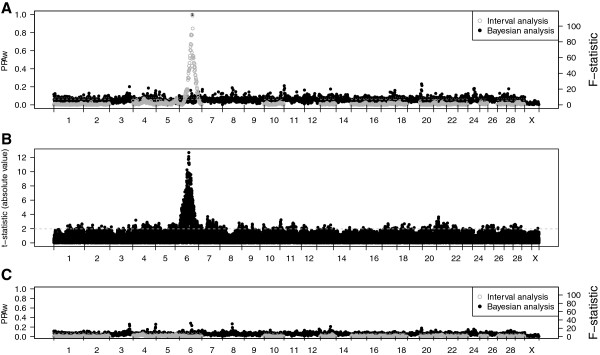
**Combined plots of QTL analyses using Btau4.0 build with interval and Bayesian methods. (A)** Original model with chromosome-wide significant thresholds (*P* = 0.01 and 0.05) as the dashed and dotted lines, respectively, for the interval analysis, while the posterior probability of association (PPA_*w*_) is plotted for the Bayesian analysis; **(B)** original model using single-marker association methods with absolute value of t-statistics and *P* = 0.05 threshold as the dashed line; **(C)** model including *KIT* region breed-of-origin genotypes with chromosome-wide significant thresholds as described before.

Unexpectedly, a major locus associated with degree of black was detected on BTA6 (*Bos taurus* chromosome 6) at an average location of 71 Mb (95% CI from 60 to 73 Mb) by interval analysis (Table 1). Suggestive (*P* < 0.05) and significant (*P* < 0.01) QTL were also identified on BTA4 and 5, respectively. When 10 of the 29 bovine chromosomes generated runtime errors due to long strings of heterozygous SNPs, single-marker association analysis was run and detected the major locus on BTA6 (Bonferroni corrected *P* = 3.88e^-23^), but did not confirm the QTL on BTA4 or 5. Bayesian association analysis also confirmed the major locus on BTA6 (but not those on BTA4 or 5) with PPA_*w*_ = 1.00 (Figure 2A-B). The posterior mean of genetic variance for the 72 Mb region accounted for 43.75% of the total genetic variance using Bayesian association analysis, and an expanded region from 68 Mb to 72 Mb region accounted for 44.19% of the total genetic variance. Posterior means of variances and genomic heritability are in Table 2.

**Table 1:** **Locations, test statistics, size of effects, and proportion of variance explained of QTL for degree of black detected using *E***^***D***^ ***E***^***+ ***^**F**_**2 **_**Nellore-Angus cattle using interval analysis**

**BTA**	**Pos (Mb)**	**Flanking markers**	**Test statistics**			**Effects**		
			**F**	**LRT**	**LOD**	**Mean ± SE**	**Additive**^**a **^**± SE**	**Dominance**^**b **^**± SE**
4^*^	59	Hapmap23995-BTA142201 - ARS-BFGL-NGS-105821	8.23	15.82	3.44	-0.5052 ± 0.1989	-0.4765 ± 0.1986	0.9234 ± 0.2834
5^**^	110	Hapmap3063-BTA-15439 - UA-IFASA-8960	8.87	17.01	3.69	0.5213 ± 0.1858	-0.0598 ± 0.1842	-1.2687 ± 0.3080
6^**^	71	ARS-BFGL-NGS-93633 - Hapmap24750-BTC-042016	114.61	153.42	33.31	-0.5183 ± 0.1326	-1.7699 ± 0.1327	1.1841 ± 0.1874

^a^QTL genotypic value of Nellore homozygote such that 2a = NN-AA; ^b^NA heterozygote deviation from QTL homozygote midpoint such that d = NA-0.5 (NN + AA); ^*^suggestive chromosome-wide (*P* < 0.05); ^**^significant chromosome-wide (*P* < 0.01).

**Table 2:** **Posterior means of parameters using Bayesian inference with**π^**= 0.995**^**1**^

**Model**^**2**^	**Estimated total phenotypic variance**	**Estimated additive genetic variance**	**Estimated residual variance**	**Estimated genomic heritability**
Fixed effects (FE)	5.1984	4.6139	0.5844	0.8860
FE with *KIT* region	2.9747	2.4582	0.5166	0.8196
FE with *PDGFRA* region	2.6959	2.2043	0.4916	0.8117
FE with *CORIN* region	2.7168	2.2564	0.4604	0.8247

^1^Posterior means from Bayesian analyses were conducted using BayesB procedures in GenSel with 51 000 iterations and the first 1000 iterations discarded as burn-in; variances are reported in squared units of degree of black; ^2^ “fixed effects” refers to the final model for degree of black, which included fixed effects of sire, family nested within sire, sex, and season of photograph; additional models including “with” indicates the inclusion of candidate region as fixed effect using the breed of origin genotype specified.

The position of the major locus on BTA6 coincided with a cluster of tyrosine kinase receptor genes including *platelet-derived growth factor receptor alpha polypeptide* (*PDGFRA*), *v-kit Hardy-Zuckerman 4 feline sarcoma viral oncogene homolog (KIT)*, and *kinase insert domain receptor* (*KDR*). Mutations in *KIT* cause pigmentation defects and patterns in several species (as reviewed by Fontanesi et al. [18]), and QTL for spotting have been found at or near *KIT* in cattle [18-20]. The frequency of any spotting (body or face) among *E*^*D*^*E*^*+*^ heterozygotes in this study was 0.2, so to confirm that the QTL detected was indeed degree of black and not a false positive association with spotting, two additional analyses were performed. First, spotting (presence or absence of any white spotting on the body or face) was fit as a fixed effect in the model. Although this effect was significant (*P* = 0.0025), the QTL for degree of black on BTA6 remained strongly significant. Second, the analysis was repeated using the subset of non-spotted animals (n = 167), and the QTL on BTA6 was detected as before. Both of these analyses confirm that degree of black and spotting are the result of separate loci and that these loci are probably tightly linked.

Because of the known association between *KIT* and coat color, breed-of-origin of a 1 Mb region that spans these three genes and centered on *KIT* was assigned for 204 of the 208 F_2_*E*^*D*^*E*^*+*^ Nellore-Angus cattle. Breed-of-origin of this region and an interaction of breed-of-origin with sire was significant (*P* < 0.05). For all sires, NN homozygotes for the *KIT* region differed (*P* < 0.05) from AA homozygotes for degree of black, and red pigmentation appeared to be recessive and was associated with the Nellore allele. For three of the sires, the NN homozygotes had less black pigmentation as compared with any alternate breed-of-origin combination (AN, NA, and AA), whereas the remaining sire had NN homozygotes different from AA homozygotes, but not AN or NA genotypes. Fitting breed-of-origin of the *KIT* region into the original model accounted for the variation attributed to the major locus on BTA6 (Figure 2C).

Modeling specific haplotype pairs instead of breed-of-origin permitted the same SNP haplotypes (and presumably the same alleles of these three tyrosine kinase receptors) to be present in both *Bos indicus* and *Bos taurus*. However no *KIT* region phased haplotypes were shared between the Nellore and Angus founders and therefore did not provide any additional information from the breed-of-origin genotypes previously described.

**Figure 3:**
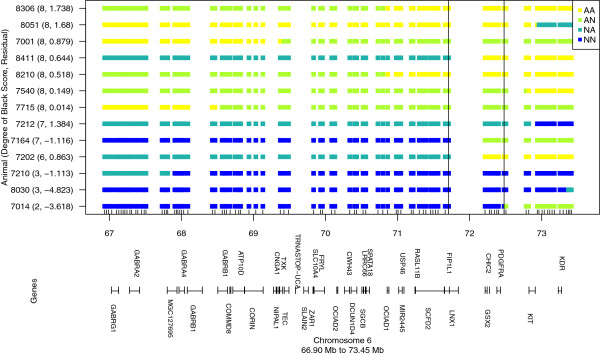
**Recombinants for a 5 Mb region that includes *CORIN*, *PDGFRA*, *KIT*, and *KDR*.** The animal identifier for each genotype (AA, AN, NA, NN where allele from sire is listed first) is followed in brackets by the raw degree of black score and the residual after adjusting for fixed effects of sex, family, and season; vertical solid black lines indicate the interval most likely to contain the reddening locus under the assumption that reddening is associated with the Nellore allele and acts as a recessive; genes within this region (excluding model genes designated as “LOC”) are plotted based on their start and end base pair positions using “|”.

Because long-range haplotypes are recovered in this population due to linkage, we considered candidate genes up to 5 Mb from the leading SNP. The gene *corin serine peptidase* (*CORIN*) is located 3.7 Mb upstream of *PDGFRA* and is associated with lighter coat colors in mice [21]. Haplotypes and breed-of-origin were assigned as before and 13 recombinants with degree of black scores were identified (Figure 3). Assuming that the reddening associated with the Nellore allele was recessive, the interval could be refined to a region between 71708424 and 72478577 bp on BTA6 (Figure 3), thereby eliminating the coding sequences of *KIT*, *KDR* and *CORIN* as candidates. Although we cannot rule out possible effects of long-range regulatory elements associated with these genes, based on its location within the refined interval, *PDGFRA* appears to be the strongest positional candidate for the reddening phenotype.

## Discussion

For the major locus on BTA6, the strongest positional candidate gene is *PDGFRA*, which is a member of the platelet-derived growth factors (PDGF) family that consists of two receptors (α and β) and four ligands (A-D). Although we are not aware of any previously documented role for *PDGFRA* in cattle coat color, its function and cellular responses have been extensively studied in the developing mouse. To function as a tyrosine kinase receptor, the PDGF-receptor must first bind a ligand, then dimerize with another ligand-bound PDGF-receptor (reviewed by Hoch and Soriano [22]). Signal transduction pathways including the Ras-mitogen activated protein kinase (Ras-MAPK), phosphatidylinositol 3-kinase (PI3K), and phospholipase Cγ pathways can be activated by both PDGF-receptors.

In relation to pigmentation, *PDGFRA* has been shown to have a role in mouse hair follicle development [23] and possibly in melanocyte migration [24] although differentiation of the roles of *PDGFRA* and *KIT* is difficult. Based on its functions described in mouse (reviewed by Hoch and Soriano [22]), *PDGFRA* could influence the MAPK signaling pathway in melanogenesis and therefore the expression of *MITF*, which controls a complex cascade of events in melanogenesis [25]. Furthermore, *PDGFRA* can activate the expression of the *protein kinase C* (*PKC*) gene and influence Ca^2+^ levels, thereby directly impacting melanin synthesis.

During melanogenesis, *KIT* drives melanocyte migration from the neural crest along the dorsolateral pathway. Mutations associated with *KIT* result in white coat color or white spotting in numerous species including cattle [18]. In 2012, it was shown that color sidedness in cattle is determined by translocations that include the *KIT* locus [26]. Although we are not aware of any previously reported association of KIT with reddening, it is possible that copy number variation in this region may be a factor and warrants further investigation.

Located approximately 3.7 Mb upsteam of *PDGFRA* is the gene *CORIN* that encodes a mosaic protein expressed primarily in the heart and necessary for pro-atrial natriuretic peptide (pro-ANP) activation. Enshell-Seijffers et al. [21] characterized *CORIN* expression in the dermal papilla of the hair follicle and found that mice homozygous for the *CORIN* mutation (C^–^/C^–^) had lighter coat colors than mice with at least one functional *CORIN*. This observation depended on the genotype at the *Agouti signaling protein* (*ASIP*) gene, where at least one wild type or functional *ASIP* allele was necessary for homozygous *CORIN* mutants to express the altered pheomelanin production. The *CORIN* mutation was associated with an increase in the length of the basal band, which contains pheomelanin. Because of this, a larger proportion of the hair shaft contained pheomelanin and therefore produced a lighter coat color than wild types. A variant agouti protein was observed in only one family in our population (data not shown), which ruled out a similar interaction in cattle as the reason for the large effect observed.

Nigrovic et al. [27] reported that a dominant spotting mutation termed W^sh^ in mice contains a genetic inversion that affects a regulatory element for *KIT*, downstream of *PDGFRA* in the 3’ breakpoint and disrupts *CORIN* expression due to the 5’ breakpoint that occurs between the fifth and sixth exons. Structural variation or other regulatory elements in the *CORIN-PDGFRA* interval cannot be ruled out based on these data and will be explored using higher density assays in the future.

## Conclusion

Suggestive and significant QTL at the chromosome-wide level were identified on BTA4 and 5 using interval analysis for degree of black, but these QTL were not confirmed by single-marker association or Bayesian methods. The major locus at 72 Mb on BTA6 accounted for 43.75% of genetic variation using BayesB procedures with π^ = 0.995. This region coincides with a cluster of *tyrosine kinase receptor* genes including *PDGFRA*, *KIT*, and *KDR*. Fitting breed-of-origin of the 1 Mb region that contains these three genes in the model accounted for the majority of variation attributed to the major locus. Therefore, one of these three coding genes, or an associated structural or regulatory variant, is probably responsible for the majority of the variation in the reddening phenotype. Recombinants within the F_2_*E*^*D*^*E*^*+*^ Nellore-Angus cattle identified *PDGFRA* as the strongest positional candidate gene for the reddening phenotype using the current marker panel, but additional positional candidates are present (e.g., *CORIN*). Further research using higher density markers and functional analyses will be required to confirm the role of *PDGFRA* or other genes, and structural or regulatory variants, and their interaction with *MC1R* for the modification of black coat color in these cattle influenced by *Bos indicus*.

## Competing interests

The authors declare that they have no competing interests.

## Authors’ contributions

JOS, DGR, and CAG contributed to the conception and design of the study. JOS, DGR, CAG, and LLHH contributed to the statistical design and interpretation of the results. JOS and LLHH collected and formatted photographs for phenotype scoring, and LLHH, JOS, and DGR scored the phenotype. CAG contributed to data formatting and interpretation of results from QTL analyses and use of breed-of-origin data. CAA performed parentage testing, contributed to sequencing and interpretation of results. LLHH contributed to QTL analyses, breed-of-origin genotypes, and interpretation of results. All authors contributed to manuscript drafts and revision. All authors read and approved the final manuscript.
